# Urinary podocalyxin as an early biomarker for diabetic nephropathy

**DOI:** 10.1371/journal.pone.0347975

**Published:** 2026-07-23

**Authors:** Md. Sabbir Hossain, Shamsia Tasnim Dwipi, Nazma Ahmed, Khaled Mahbub Murshed, Kazi Ali Aftab, Abdullah Al Mahdi, Md. Mojibul Hoque, Md. Jobayer Hossain Taraq, Md. Zakir Hussain, Md. Abul Kalam Azad

**Affiliations:** 1 Department of Internal Medicine, Bangabandhu Sheikh Mujib Medical University, Dhaka, Bangladesh; 2 National Institute of Cardiovascular Diseases, Dhaka, Bangladesh; Guangdong Nephrotic Drug Engineering Technology Research Center, Institute of Consun Co. for Chinese Medicine in Kidney Diseases, CHINA

## Abstract

**Background:**

Conventional biomarkers, such as the urinary albumin-to-creatinine ratio (uACR), detect diabetic nephropathy (DN) late in its course and may miss early podocyte injury. Urinary podocalyxin (u-PDX), a sialylated glycoprotein shed from podocytes, may offer an earlier means of detection.

**Methods:**

We conducted a cross-sectional analytical study at Bangabandhu Sheikh Mujib Medical University between March 2023 and August 2024. Eighty-eight adults were enrolled: 59 participants with type 2 diabetes mellitus (T2DM) and 29 healthy controls. Participants with diabetes were stratified into normoalbuminuric (n = 29), microalbuminuric (n = 15), and macroalbuminuric (n = 15) subgroups based on uACR. Clinical variables and biochemical indices were recorded. u-PDX concentrations were measured using ELISA, and associations with uACR and eGFR were analyzed using Kendall’s tau-b and Pearson’s correlation coefficients. Diagnostic performance was assessed using receiver operating characteristic (ROC) curves with bootstrap internal validation.

**Results:**

Among participants with diabetes, u-PDX levels increased progressively across normo-, micro-, and macroalbuminuric groups (p < 0.001). u-PDX correlated positively with fasting blood glucose, post-glucose plasma glucose, HbA1c, serum creatinine, serum urea, and uACR, with the strongest correlation for uACR (r = 0.79, p < 0.001), and inversely with serum albumin (r = −0.62, p < 0.001) and eGFR (r = −0.51, p < 0.001). In pairwise ROC analysis, a u-PDX cutoff of 0.80 ng/mL discriminated healthy controls from diabetic patients without albuminuria (sensitivity 100.0%, specificity 96.4%); 1.60 ng/mL discriminated diabetic patients without albuminuria from those with microalbuminuria (86.7%, 93.1%); and 5.51 ng/mL discriminated microalbuminuric from macroalbuminuric patients (86.7%, 93.3%). For overall discrimination of albuminuria-defined DN among diabetic patients, the optimal u-PDX cutoff was 2.92 ng/mL (AUC 0.96, 95% CI 0.90–1.00).

**Conclusions:**

Urinary podocalyxin is a sensitive early biomarker of diabetic nephropathy, rising before overt albuminuria and correlating with albuminuria severity and declining eGFR. However, longitudinal validation is required before u-PDX can be recommended for routine screening in adults with T2DM.

## 1. Introduction

Chronic kidney disease (CKD) represents a significant global health challenge, affecting more than 10% of the population and over 800 million people worldwide (1). Because the prevalence of diabetes continues to rise, CKD imposes a substantial economic burden, and its associated mortality has increased despite advances in therapy [1]. People living with diabetes and hypertension constitute a large proportion of those affected by CKD [2], and diabetic nephropathy remains the primary driver of progression to end-stage renal disease (ESRD) [3]. Diabetic nephropathy is characterized by a long, clinically silent phase during which structural glomerular and podocyte injury accumulates before conventional markers become abnormal; consequently, recognizing patients at elevated risk at an early stage is essential to slow progression and prevent advancement to ESRD.

In routine practice, assessment for diabetic nephropathy typically relies on the urine albumin-to-creatinine ratio (uACR) alongside the estimated glomerular filtration rate (eGFR). Microalbuminuria, defined as 30–300 mg/g of creatinine, is regarded as the benchmark indicator for early diabetic nephropathy and a strong predictor of progression to ESRD [4]. However, albumin excretion fluctuates with exercise, diet, infection, and blood pressure, requiring repeated testing [5]. Large population surveys have shown discordance between albuminuria and renal function; in the NHANES III cohort, 36% of diabetic patients with advanced CKD had normal uACR, and only approximately half of those with renal impairment had preceding albuminuria [6]. Some patients lose kidney function before microalbuminuria appears, while others revert from microalbuminuria to normoalbuminuria [7]. These observations indicate that albuminuria reflects glomerular injury but cannot reliably detect the earliest stages of DN [8], prompting the search for more sensitive biomarkers. An accurate, universally accepted early biomarker for diabetic nephropathy remains unavailable, supporting the rationale for investigating podocyte injury markers.

Podocyte injury is central to the pathogenesis of diabetic glomerulopathy. Podocytes are highly specialized, terminally differentiated epithelial cells whose interdigitating foot processes cover the glomerular basement membrane and play a critical role in preserving the selectivity of the filtration barrier [9]. In diabetes, podocytes become hypertrophic, foot processes broaden and detach, and the number and density of podocytes decline (podocytopenia) [10]. Because podocytes sit on the external side of the basement membrane, apical injury leads to the shedding of viable cells or vesicles into the urinary space [2]. These fragments contain podocyte-specific proteins that are accessible via non-invasive urinary assays and may signal glomerular injury before overt albuminuria.

Hara et al. demonstrated that podocalyxin, a heavily sialylated glycoprotein on the podocyte surface, can be detected in urine using a highly sensitive ELISA [11]. In their cohort, more than half of normoalbuminuric diabetic individuals and approximately two-thirds of those with micro- or macroalbuminuria had urinary podocalyxin levels above the predefined threshold, and these levels correlated with glycemic control and tubular injury but not with serum creatinine or eGFR [11], implying that urinary podocalyxin reflects early podocyte damage. In a cross-sectional analysis of 90 diabetic participants and 30 healthy controls, Kostovska et al. observed that urinary podocalyxin concentrations rose steadily with worsening albuminuria, showed a modest correlation with uACR, and identified a threshold of 43.8 ng/mL yielding 73.3% sensitivity and 93.3% specificity for detecting diabetic nephropathy [12]. This marker identified elevated podocyte injury in nearly half of normoalbuminuric patients, whereas the conventional microalbumin cut-off detected only 41.5% of cases, leading the authors to propose urinary podocalyxin as a more sensitive and specific marker for early diabetic nephropathy [12]. Sensitive biomarkers therefore have a dual role: improving diagnosis and enabling earlier, potentially modifiable intervention.

Beyond detection, early identification of at-risk individuals is clinically valuable because it opens a window for early lifestyle and dietary intervention. Interest in dietary and “food and medicine homology” approaches as adjuncts in the prevention and management of diabetes and its renal complications has grown considerably [13]. Although previous studies suggest that urinary podocalyxin may serve as an early marker of podocyte injury, uncertainties remain. Reported diagnostic thresholds vary substantially, correlations with renal function indicators are inconsistent, and evidence for generalizability across populations is limited. These gaps are particularly relevant for South Asian populations, where the prevalence of diabetes and renal complications is markedly high. Furthermore, the capacity of urinary podocalyxin to detect early podocyte damage in normoalbuminuric individuals has not been firmly established, underscoring the need for validation in ethnically diverse settings.

Against this background, the present study was conducted among Bangladeshi adults with diabetes across stages of albuminuria, alongside a comparison group of healthy individuals. By evaluating u-PDX, uACR, and related clinical parameters, this study aimed to determine the diagnostic value of u-PDX as an early indicator of diabetic nephropathy and to compare its performance with that of conventional microalbuminuria, thereby informing the identification of populations who might benefit from early intervention.

## 2. Methods

### 2.1 Study design

This cross-sectional analytical study examined the discriminatory capacity of urinary podocalyxin for identifying diabetic nephropathy in adults with T2DM. This design was selected because it enables concurrent evaluation of the biomarker and the clinical outcome (albuminuria-based DN status) within a defined study population. We acknowledge at the outset that a cross-sectional design cannot establish causality or prognostic value.

### 2.2 Study setting

Participants were enrolled from both inpatient and outpatient services of the Department of Internal Medicine at Bangabandhu Sheikh Mujib Medical University (BSMMU), Dhaka, Bangladesh. Recruitment and data collection occurred between 25 March 2023 and 3 August 2024. All samples were collected and processed using BSMMU clinical laboratories and associated facilities. As a single-center study, the setting is reflected in the interpretation of generalizability.

### 2.3 Sample size and sampling

The total sample comprised 88 participants. Given the limited prior regional data on urinary podocalyxin and the exploratory nature of the study, a pragmatic recruitment target of approximately 30 participants in each major study category was adopted, consistent with previous pilot work [14]. Accordingly, we recruited 29 patients with diabetes without albuminuria, 30 patients with diabetes and albuminuria, and 29 healthy controls. The albuminuric diabetic cohort was subsequently stratified into microalbuminuria and macroalbuminuria subgroups. Participants were recruited using consecutive sampling: eligible patients attending BSMMU or affiliated primary health-care centers were approached sequentially, while healthy controls were recruited from hospital staff and relatives accompanying outpatients. Recruitment continued until the prespecified target was achieved.

### 2.4 Eligibility criteria

Eligible participants were adults aged ≥ 40 years. Individuals in the diabetic nephropathy group were adults with confirmed type 2 diabetes who showed signs of kidney damage according to KDIGO 2012 criteria, including raised serum creatinine, reduced eGFR, and albuminuria (uACR ≥ 30 mg/g). Individuals with T2DM but without DN were included if they were newly diagnosed, demonstrated no micro- or macroalbuminuria, and had no evidence of other renal disease. Healthy controls were individuals without diabetes, hypertension, or renal disease. Diabetes duration was recorded for all diabetic participants but was not applied as an eligibility threshold. All participants provided written informed consent prior to enrollment.

Exclusion criteria comprised the use of renin–angiotensin system (RAS) inhibitors, including angiotensin-converting enzyme inhibitors and angiotensin receptor blockers, as well as rate-limiting calcium channel blockers. Patients receiving any of these agents were not enrolled; consequently, no participant in the study was taking these medications, and no washout period was applied or required. Participants were additionally excluded if they had secondary causes of renal impairment (e.g., glomerulonephritis, urinary tract infection, pregnancy, uncontrolled hypertension, acute febrile illness, or alcoholism at the time of enrollment), or CKD attributable to non-diabetic etiologies. Common metabolic comorbidities such as hyperlipidemia and gout were not prespecified exclusion criteria; this is acknowledged as a potential source of residual confounding in the limitations.

### 2.5 Classification of participants

Diabetic participants were categorized according to uACR from a spot urine specimen, in accordance with KDIGO 2012 guidelines. Individuals with normoalbuminuria (uACR < 30 mg/g; n = 29) were classified as having no diabetic nephropathy, whereas those with microalbuminuria (uACR 30–300 mg/g; n = 15) and macroalbuminuria (uACR > 300 mg/g; n = 15) were grouped as having diabetic nephropathy. For analytical purposes, participants with micro- and macroalbuminuria were classified as having DN, while normoalbuminuric diabetics and healthy controls were categorized as non-DN.

### 2.6 Variables and data collection

We collected sociodemographic data (age, sex, religion, education, occupation) and clinical history (body mass index, duration of diabetes, blood pressure). Laboratory variables included fasting blood sugar, 2-hour post-prandial glucose, HbA1c, serum creatinine, serum urea, serum albumin, total protein, eGFR, urinary albumin, urinary creatinine, and u-PDX. All procedures were performed by trained nurses or laboratory technicians blinded to participant group.

#### 2.6.1 Urine sampling and storage.

Participants provided a 10 mL mid-stream spot urine sample in a sterile container without preservatives. Each sample was immediately tested by dipstick for proteinuria and glucose, then divided into one aliquot for microalbumin and creatinine measurement and another for u-PDX analysis. Before storage, urine was centrifuged at approximately 1,000 × g for 20 minutes to remove cellular material and particulate debris, and the supernatant was stored at −80 °C until analysis. Each sample was assigned an anonymized identification code.

#### 2.6.2 Measurement of albumin and creatinine.

Urinary microalbumin was quantified using a turbidimetric immunoassay on an Atellica Solution analyzer (Siemens, USA). Urinary creatinine was measured using the Jaffe reaction on a ChemWell 2910 biochemical analyzer (Awareness Technology, USA). Calibration and quality-assurance procedures followed the manufacturers’ recommended protocols.

#### 2.6.3 Measurement of u-PDX.

u-PDX was quantified using a commercially available enzyme-linked immunosorbent assay (ELISA) kit (Exocell, Philadelphia, PA, USA). Samples were diluted 1:2 in assay buffer and analyzed in duplicate using an indirect competitive ELISA, in which polyclonal anti-podocalyxin antibodies in the sample compete with plate-bound podocalyxin, and bound antibody is detected with a horseradish peroxidase–conjugated secondary antibody. Optical density was read at 450 nm, and u-PDX concentrations (ng/mL) were derived from a standard curve generated with known podocalyxin standards. Both intra-assay and inter-assay coefficients of variation were below 8%, in accordance with the manufacturer’s specifications.

#### 2.6.4 Blood sampling and biochemical analyses.

After an overnight fast, venous blood was collected from the antecubital vein into EDTA and plain tubes. Samples were centrifuged at approximately 1,000 × g for 10 minutes to obtain plasma or serum. Fasting plasma glucose, postprandial plasma glucose, HbA1c, urea, creatinine, total protein, and albumin were analyzed using enzymatic methods on a ChemWell 2910 automated analyzer. HbA1c was quantified from EDTA whole blood using high-performance liquid chromatography. Estimated glomerular filtration rate (eGFR) values (mL/min/1.73 m²) were reported directly by the clinical laboratory.

### 2.7 Outcome assessment and diagnostic accuracy

Our primary outcome was the presence of DN, defined according to the KDIGO albuminuria categories. To evaluate diagnostic performance, we compared u-PDX concentrations across groups and plotted ROC curves to discriminate (a) healthy controls from diabetic patients without albuminuria, (b) diabetic patients without albuminuria from those with microalbuminuria, and (c) microalbuminuric from macroalbuminuric diabetic patients. We calculated areas under the ROC curve (AUC), sensitivity, specificity, and Youden’s J index to determine optimal cut-offs. To assess potential over-optimism, ROC analyses were internally validated using bootstrap optimism correction with 2,000 resamples, and optimism-corrected AUC estimates were calculated for each pairwise comparison.

### 2.8 Statistical analysis

Data were entered into a secure database and analyzed using IBM SPSS Statistics version 27 (IBM Corp., Armonk, NY, USA), with penalized regression performed in R. Continuous variables were summarized as mean ± standard deviation or median (interquartile range), as appropriate, and categorical variables as counts and percentages. Normality was assessed using the Shapiro–Wilk test. Group comparisons used one-way analysis of variance (ANOVA) or Kruskal–Wallis tests, followed by post-hoc comparisons with Bonferroni correction. Trends across ordered albuminuria categories were assessed using the Jonckheere–Terpstra test. Associations between u-PDX and continuous variables were assessed using Pearson’s correlation coefficient for variables meeting approximate linearity and normality assumptions, and Kendall’s tau-b as a rank-based, distribution-robust measure suitable for non-normally distributed and ordinal data; both are reported for transparency. The independent association between u-PDX and DN was examined using logistic regression adjusted for age, sex, duration of diabetes, and systolic blood pressure. Because the strong separation between groups produced unstable maximum-likelihood estimates (quasi-complete separation), the model was fitted using Firth’s penalized likelihood, and adjusted odds ratios (aOR) are reported per 1 ng/mL increase in u-PDX with 95% confidence intervals. Statistical significance was set at p < 0.05. Missing data were minimal (< 2%) and handled using listwise deletion.

### 2.9 Ethical considerations

The study was approved by the Institutional Review Board of BSMMU (BSMMU/2023/3056), which operates within Bangladesh’s national ethical framework for research involving human participants. All procedures complied with the Declaration of Helsinki. Participants provided written informed consent after receiving information about the study objectives, procedures, potential risks, and benefits; informed consent was not waived. To protect confidentiality, each participant and biological sample was assigned a coded identification number, with personal identifiers stored separately and excluded from the analysis dataset.

## 3. Results

### 3.1 Participant characteristics

**Fig 1:**
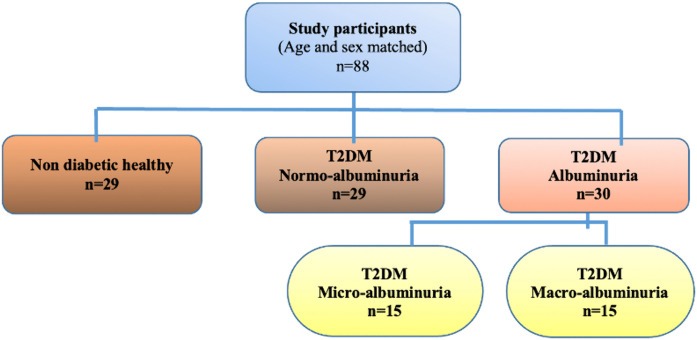
Flow diagram of study participants and classification. Eighty-eight age- and sex-matched participants comprised 29 non-diabetic healthy controls and 59 participants with type 2 diabetes mellitus (T2DM), the latter divided into normoalbuminuria (n = 29) and albuminuria (n = 30); the albuminuric group was further stratified into microalbuminuria (n = 15) and macroalbuminuria (n = 15).

A total of 88 individuals were analyzed, comprising 59 participants with T2DM and 29 healthy participants. Among those with diabetes, 29 had normoalbuminuria, 15 had microalbuminuria, and 15 had macroalbuminuria, yielding 30 participants classified as having diabetic nephropathy (Table 1, Fig 1). The mean age of the cohort was 53.6 ± 10.6 years, with no significant age differences between groups (p > 0.05). The duration of type 2 diabetes was numerically higher with increasing albuminuria severity (normoalbuminuria 6.6 ± 5.3, microalbuminuria 8.3 ± 5.0, macroalbuminuria 9.3 ± 5.2 years; overall 7.7 ± 5.2 years among diabetic participants), although this trend did not reach statistical significance (p = 0.15). Males comprised 69.3% of the sample, with the highest proportion in the macroalbuminuric (80.0%) and normoalbuminuric (79.3%) groups and lower in the microalbuminuric group (46.7%); 65.5% of healthy controls were men. Education and occupation distributions are shown in Table 1, with no clear group differences.

**Table 1:** Demographic characteristics of diabetic and healthy participants (n = 88).

Parameter	Total (n = 88)	Macroalb. (n = 15)	Microalb. (n = 15)	Normoalb. (n = 29)	Healthy (n = 29)
Age (years)	53.6 ± 10.6	59.5 ± 12.5	54.2 ± 11.4	51.0 ± 8.95	52.9 ± 9.98
Duration of T2DM (years)†	7.7 ± 5.2	9.3 ± 5.2	8.3 ± 5.0	6.6 ± 5.3	N/A
Sex, Male / Female	61 (69.3) 27 (30.7)	12 (80) 3 (20)	7 (46.7) 8 (53.3)	23 (79.3) 6 (20.7)	19 (65.5) 10 (34.5)
Education: Below SSC / SSC–HSC / Graduate	32 (36.3) 17 (19.3) 39 (44.4)	7 (46.7) 4 (26.7) 4 (26.7)	7 (46.7) 3 (20) 5 (33.3)	11 (37.9) 6 (20.7) 12 (41.4)	7 (24.1) 4 (13.8) 18 (62.1)
Occupation: Service / Business / Retired / Housewife / Other	32 (36.4) 17 (19.3) 12 (13.6) 17 (19.3) 10 (11.4)	5 (33.3) 4 (26.7) 3 (20) 2 (13.3) 1 (6.7)	4 (26.7) 3 (20) 1 (6.7) 4 (26.7) 3 (20)	12 (41.2) 4 (13.7) 4 (13.7) 6 (20.1) 3 (10.3)	11 (38) 6 (20.7) 4 (13.8) 5 (17.2) 3 (10.3)

*Data presented as frequency (percentage) per column; age and duration as mean ± SD. †Duration of diabetes is reported for participants with diabetes; the Total column reflects all diabetic participants and is not applicable (N/A) to healthy controls. Kruskal–Wallis comparison of duration across diabetic subgroups: p = 0.15. SSC, Secondary School Certificate; HSC, Higher Secondary Certificate.*

### 3.2 Clinical and biochemical parameters according to albuminuria status

**Fig 2:**
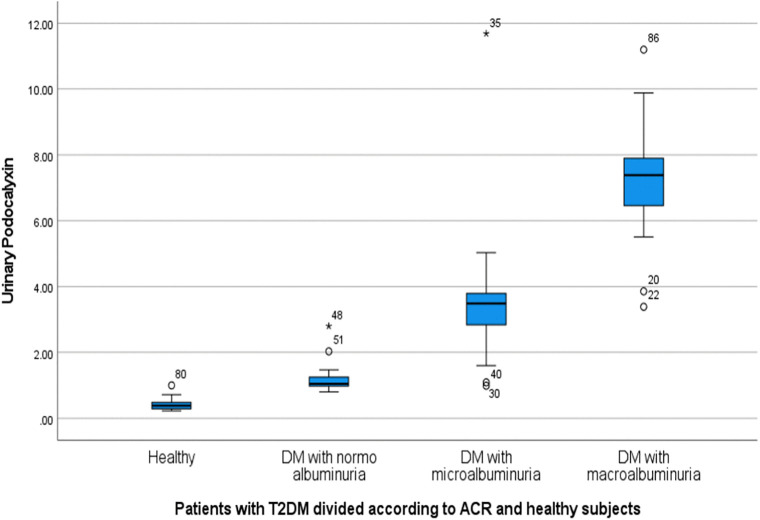
Urinary podocalyxin (u-PDX) concentrations across albuminuria categories. Box plots show the median, interquartile range, and outliers for healthy controls and T2DM patients with normo-, micro-, and macroalbuminuria; values therefore differ in presentation from the mean ± SD reported in Table 2. u-PDX increased progressively with worsening albuminuria (p < 0.001).

Table 2 summarizes clinical and biochemical parameters across groups. Body mass index, systolic blood pressure, and diastolic blood pressure did not differ significantly among groups (p > 0.05). Glycemic and renal indices showed a graded pattern across albuminuria categories. Fasting blood glucose and 2-hour post-glucose levels were markedly higher in all diabetic subgroups than in controls (p < 0.001). HbA1c followed a similar pattern (9.41 ± 1.55% in normoalbuminuria vs. 8.65 ± 2.83% in macroalbuminuria; p < 0.001). Serum creatinine and urea increased progressively with albuminuria severity: creatinine rose from 0.97 ± 0.19 mg/dL in normoalbuminuric diabetics to 1.64 ± 1.08 mg/dL in macroalbuminuric patients, and urea from 30.70 ± 12.74 to 47.70 ± 24.67 mg/dL (both p < 0.001). Serum albumin decreased from 4.47 ± 0.30 to 3.76 ± 0.60 g/dL (p < 0.001), and eGFR declined from 85.69 ± 18.40 to 62.61 ± 33.18 mL/min/1.73 m² (p < 0.001). Mean uACR increased sharply from 8.39 ± 4.28 to 1,561.01 ± 1,157.76 mg/g, and u-PDX concentrations increased from 1.16 ± 0.39 to 7.28 ± 2.05 ng/mL across the same categories (p < 0.001). Fig 2 illustrates the progressive increase in u-PDX with worsening albuminuria.

**Table 2:** Clinical and laboratory data among T2DM subgroups by ACR and healthy subjects.

Variable	Healthy	T2DM with normoalbuminuria	T2DM with microalbuminuria	T2DM with macroalbuminuria	p-value
Age	52.86 ± 9.97	50.97 ± 8.94	54.20 ± 11.42	59.47 ± 12.50	0.11
BMI	24.81 ± 2.54	25.37 ± 3.22	25.01 ± 3.01	24.91 ± 4.67	0.94
SBP	120.86 ± 8.67	121.38 ± 11.87	119.0 ± 9.29	121.33 ± 12.02	0.90
DBP	77.24 ± 5.27	75.17 ± 6.87	74.00 ± 6.32	75.0 ± 6.27	0.35
FBS	4.98 ± 0.42	9.97 ± 4.08	9.18 ± 4.18	9.25 ± 4.70	<0.001
ABF	5.72 ± 1.04	18.34 ± 5.72	15.61 ± 6.38	13.53 ± 7.70	<0.001
HbA1c	5.44 ± 0.54	9.41 ± 1.55	9.24 ± 2.34	8.65 ± 2.83	<0.001
S. Creatinine	0.72 ± 0.16	0.97 ± 0.19	1.14 ± 0.59	1.64 ± 1.08	<0.001
S. Urea	21.9 ± 4.94	30.70 ± 12.74	33.99 ± 13.57	47.70 ± 24.67	<0.001
S. Protein	7.30 ± 0.35	7.31 ± 0.62	7.48 ± 0.73	6.98 ± 0.51	0.09
S. Albumin	4.62 ± 0.29	4.47 ± 0.30	3.93 ± 0.53	3.76 ± 0.60	<0.001
eGFR	112.24 ± 23.54	85.69 ± 18.40	77.93 ± 38.97	62.61 ± 33.18	<0.001
ACR	5.47 ± 4.69	8.39 ± 4.28	90.80 ± 51.68	1561.01 ± 1157.76	<0.001
u-PDX	0.42 ± 0.17	1.16 ± 0.39	3.69 ± 2.50	7.28 ± 2.05	<0.001

*BMI, body mass index; SBP/DBP, systolic/diastolic blood pressure; FBS, fasting blood sugar; ABF, after-breakfast (2-hour post-glucose) blood sugar; HbA1c, glycated hemoglobin; eGFR, estimated glomerular filtration rate (mL/min/1.73 m²); ACR, albumin-to-creatinine ratio; u-PDX, urinary podocalyxin.*

### 3.3 Comparison of diabetics with and without diabetic nephropathy

When participants with diabetes were reclassified as with or without DN, similar patterns were observed (Table 3). Serum creatinine, urea, and uACR were significantly higher in albuminuric diabetics (1.38 ± 0.89 mg/dL, 40.84 ± 20.77 mg/dL, and 825.91 ± 1,098.82 mg/g, respectively) than in non-albuminuric diabetics (0.97 ± 0.19 mg/dL, 30.70 ± 12.74 mg/dL, and 8.39 ± 4.28 mg/g). The eGFR was correspondingly lower in albuminuric patients (70.27 ± 36.41 vs. 85.69 ± 18.40 mL/min/1.73 m²; p < 0.001). The mean u-PDX concentration was nearly five-fold higher in patients with albuminuria (5.48 ± 2.89 ng/mL) than in those without (1.16 ± 0.39 ng/mL; p < 0.001).

**Table 3:** Clinical and laboratory data by diagnosed DN status and healthy subjects.

Variable	Healthy	T2DM without albuminuria	T2DM with albuminuria	p-value
Age	52.86 ± 9.97	50.97 ± 8.94	56.83 ± 12.06	0.11
BMI	24.81 ± 2.54	25.37 ± 3.22	24.96 ± 3.86	0.88
SBP	120.86 ± 8.67	121.38 ± 11.87	120.17 ± 10.63	0.90
DBP	77.24 ± 5.27	75.17 ± 6.87	74.5 ± 6.21	0.90
FBS	4.98 ± 0.42	9.97 ± 4.08	9.21 ± 4.37	<0.001
ABF	5.72 ± 1.04	18.34 ± 5.72	14.57 ± 7.03	<0.001
HbA1c	5.44 ± 0.54	9.41 ± 1.55	8.94 ± 2.57	<0.001
S. Creatinine	0.72 ± 0.16	0.97 ± 0.19	1.38 ± 0.89	<0.001
S. Urea	21.9 ± 4.94	30.70 ± 12.74	40.84 ± 20.77	<0.001
S. Protein	7.30 ± 0.35	7.31 ± 0.62	7.23 ± 0.67	0.82
S. Albumin	4.62 ± 0.29	4.47 ± 0.30	3.84 ± 0.56	<0.001
eGFR	112.24 ± 23.54	85.69 ± 18.40	70.27 ± 36.41	<0.001
ACR	5.47 ± 4.69	8.39 ± 4.28	825.91 ± 1098.82	<0.001
u-PDX	0.42 ± 0.17	1.16 ± 0.39	5.48 ± 2.89	<0.001

*BMI, body mass index; SBP/DBP, systolic/diastolic blood pressure; FBS, fasting blood sugar; ABF, after-breakfast (2-hour post-glucose) blood sugar; HbA1c, glycated hemoglobin; eGFR, estimated glomerular filtration rate (mL/min/1.73 m²); ACR, albumin-to-creatinine ratio; u-PDX, urinary podocalyxin*

### 3.4 Correlation of u-PDX with clinical and laboratory variables

u-PDX was strongly and positively associated with multiple glycemic and renal markers (Table 4). Pearson’s coefficients showed moderate-to-strong positive correlations between u-PDX and fasting blood glucose (r = 0.49), post-glucose plasma glucose (r = 0.40), HbA1c (r = 0.42), serum creatinine (r = 0.50), serum urea (r = 0.52), and, most notably, uACR (r = 0.79), all with p < 0.001. u-PDX showed strong inverse associations with serum albumin (r = −0.62) and eGFR (r = −0.51), both p < 0.001. Age showed a borderline positive correlation (r = 0.22, p = 0.05). No significant correlations were observed with BMI or blood pressure. Kendall’s tau-b coefficients were concordant in direction and significance with the Pearson estimates (Table 4). In the Firth penalized logistic regression adjusted for age, sex, duration of diabetes, and systolic blood pressure, each 1 ng/mL increase in u-PDX was independently associated with diabetic nephropathy (aOR 22.67, 95% CI 2.47–208.09; p = 0.006); no other covariate was independently associated with the outcome (S1 Table).

**Table 4:** Correlation of u-PDX with clinical and laboratory variables.

Variable	Kendall’s τ	p-value	Pearson’s r	p-value
Age	0.15	0.04	0.22	0.05
BMI	0.02	0.81	0.02	0.86
SBP	0.04	0.56	0.07	0.50
DBP	−0.08	0.31	−0.01	0.92
FBS	0.33	<0.001	0.49	<0.001
ABF	0.31	<0.001	0.40	<0.001
HbA1c	0.38	<0.001	0.42	<0.001
S. Creatinine	0.35	<0.001	0.50	<0.001
S. Urea	0.36	<0.001	0.52	<0.001
S. Protein	−0.03	0.72	−0.08	0.45
S. Albumin	−0.42	<0.001	−0.62	<0.001
eGFR	−0.36	<0.001	−0.51	<0.001
ACR	0.61	<0.001	0.79	<0.001

*BMI, body mass index; SBP/DBP, systolic/diastolic blood pressure; FBS, fasting blood sugar; ABF, after-breakfast (2-hour post-glucose) blood sugar; HbA1c, glycated hemoglobin; eGFR, estimated glomerular filtration rate (mL/min/1.73 m²); ACR, albumin-to-creatinine ratio; u-PDX, urinary podocalyxin*

### 3.5 Diagnostic performance of u-PDX

**Fig 3:**
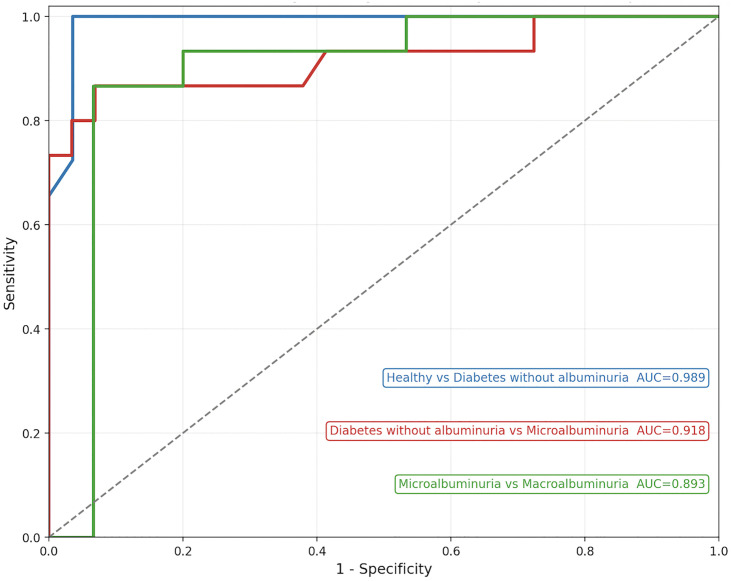
Receiver operating characteristic (ROC) curves for pairwise discrimination by urinary podocalyxin: healthy controls versus diabetes without albuminuria (AUC 0.989), diabetes without albuminuria versus microalbuminuria (AUC 0.918), and microalbuminuria versus macroalbuminuria (AUC 0.893).

Two complementary ROC analyses were performed. First, in pairwise stage comparisons, a u-PDX cutoff of 0.80 ng/mL discriminated healthy controls from diabetic patients without albuminuria with 100.0% sensitivity and 96.4% specificity; a cutoff of 1.60 ng/mL discriminated diabetic patients without albuminuria from those with microalbuminuria with 86.7% sensitivity and 93.1% specificity; and a cutoff of 5.51 ng/mL discriminated microalbuminuric from macroalbuminuric diabetic patients with 86.7% sensitivity and 93.3% specificity (Fig 3). Bootstrap internal validation demonstrated minimal optimism, with optimism-corrected AUCs remaining very close to the apparent AUCs across all pairwise comparisons (S2 Table).

Second, we evaluated overall discrimination of albuminuria-defined diabetic nephropathy from normoalbuminuric diabetes among participants with T2DM (Table 5). Because DN status in this study was defined using uACR thresholds, the ROC performance of uACR reflects incorporation of the reference-defining variable into the outcome definition and should be interpreted as an internal classification reference rather than as an independent diagnostic comparator. As expected, uACR showed perfect discrimination, with an AUC of 1.00 and 100.0% sensitivity and specificity at a cutoff of 34.06 mg/g. Against this albuminuria-defined outcome, u-PDX demonstrated excellent discriminatory performance, with an AUC of 0.96 (95% CI 0.90–1.00). The optimal u-PDX cutoff was 2.92 ng/mL, yielding 86.7% sensitivity, 100.0% specificity, 100.0% positive predictive value, 89.9% negative predictive value, and 93.2% overall accuracy. These findings indicate that u-PDX is strongly associated with albuminuria-defined diabetic nephropathy, although its diagnostic performance should be validated against longitudinal renal outcomes or independently adjudicated diabetic kidney disease in future studies.

**Table 5:** Diagnostic performance of uACR and u-PDX for diabetic nephropathy among participants with T2DM (n = 59).

Parameter	uACR	u-PDX
Area under the ROC curve (AUC)	1.00	0.96
95% confidence interval	1.00–1.00	0.90–1.00
Significance level p (area = 0.5)	<0.001	<0.001
Youden index	1.00	0.87
Optimal cutoff	34.06 mg/g	2.92 ng/mL
Total population	59	59
True positive (TP)	30	26
True negative (TN)	29	29
Sensitivity (%)	100	86.7
Specificity (%)	100	100
PPV (%)	100	100
NPV (%)	100	89.9
Accuracy (%)	100	93.2

*Diabetic nephropathy was defined using uACR thresholds; consequently, uACR is the variable defining the outcome (incorporation reference) and necessarily shows perfect discrimination. The uACR ROC is therefore presented as an internal classification reference rather than as an independent diagnostic comparator. PPV, positive predictive value; NPV, negative predictive value.*

## 4. Discussion

We measured urinary podocalyxin (u-PDX) to evaluate its utility as an early marker of diabetic nephropathy. u-PDX concentrations were markedly higher in individuals with diabetes than in healthy controls and increased progressively from the normoalbuminuric stage to microalbuminuria and macroalbuminuria. ROC analysis demonstrated progressive discriminatory performance of u-PDX across disease stages, and u-PDX correlated strongly with uACR (r = 0.79) and inversely with eGFR (r = −0.51), supporting its association with glomerular injury.

### 4.1 Comparison with previous studies

Hara et al. first established a highly sensitive ELISA for urinary podocalyxin and reported that u-PDX was above the predefined threshold in 53.8%, 64.7%, and 66.7% of normoalbuminuric, microalbuminuric, and macroalbuminuric diabetic patients, respectively [11]. In their cohort, u-PDX correlated with HbA1c and tubular injury markers but not with serum creatinine or eGFR [11]. Our findings likewise demonstrate that u-PDX increases before overt albuminuria and is more closely linked to glomerular pathology than to overall renal function; the moderate inverse correlation with eGFR observed here may reflect the later stages of podocyte loss captured in our cohort. Kostovska et al. reported a stepwise increase in u-PDX with advancing DN severity and a modest correlation with uACR (r = 0.227, p = 0.002), with a threshold of 43.8 ng/mL achieving 73.3% sensitivity and 93.3% specificity [12].

The absolute u-PDX thresholds in our cohort were lower than those previously reported and appeared stage-dependent rather than defined by a single universal cut-off. This likely reflects differences between populations, including possible ethnic and genetic variation in podocyte turnover and baseline shedding, together with assay-specific calibration, variation in ELISA methodology, and differences in the operational definition of albuminuria. In our pairwise analysis, the optimal threshold separating normoalbuminuric diabetes from microalbuminuria was 1.60 ng/mL, whereas the optimal threshold for discriminating overall albuminuria-defined DN among diabetic patients was 2.92 ng/mL (Table 5). These two values correspond to two distinct analyses (stage transition versus overall DN classification) and should not be conflated; the difference highlights that a single fixed cut-off is unlikely to be appropriate across all stages and populations.

The largest study to date involving biopsy-proven diabetic kidney disease evaluated both urinary and intrarenal podocalyxin. Zeng et al. found that urinary podocalyxin correlated with corresponding mRNA expression and with the extent of proteinuria, but urinary levels alone did not predict renal outcomes; intrarenal podocalyxin expression was an independent predictor of dialysis-free survival and more accurately reflected glomerulosclerosis [15]. Our cross-sectional design precludes assessment of prognostic value; however, the positive relationship between u-PDX and albuminuria supports the interpretation that apical podocyte injury is captured by urinary podocalyxin.

In addition to podocalyxin, other podocyte-derived markers such as nephrin and podocin have been investigated. Kostovska et al. found nephrinuria in all patients with macroalbuminuria, 88% of those with microalbuminuria, and 82% of normoalbuminuric individuals, with a strong inverse correlation with eGFR (r = −0.54) and excellent diagnostic performance (ROC-AUC 0.96) [12]. Tubular and stress-related markers, including neutrophil gelatinase-associated lipocalin (NGAL), kidney injury molecule-1 (KIM-1), N-acetyl-β-D-glucosaminidase, and transferrin, also show promise for early detection [2]. Compared with these markers, podocalyxin offers the advantage of directly reflecting podocyte-specific apical injury, whereas NGAL and KIM-1 predominantly reflect tubular rather than glomerular damage and are less specific to the podocytopathy that characterizes early diabetic glomerulopathy; conversely, many of these alternatives lack specificity or require complex assays, which limits routine use, and head-to-head comparative data remain scarce. More broadly, urinary and tissue biomarkers are increasingly applied across diverse causes of kidney injury, including environmental and drug-induced nephrotoxicity, frequently using integrative computational and single-cell approaches [16,17]; these developments underscore both the promise and the methodological challenges of biomarker-based nephrology.

### 4.2 Biological interpretation

Podocytes form the visceral component of the glomerular filtration barrier, anchoring to the glomerular basement membrane and maintaining selective permeability through slit-diaphragm complexes and an apical glycocalyx [18]. Chronic hyperglycemia and associated metabolic stressors induce podocyte hypertrophy, foot-process effacement, detachment, and apoptosis, ultimately producing podocytopenia and glomerulosclerosis [19]. Because podocytes reside on the urinary aspect of the basement membrane, apical injury releases viable cells or membrane-derived fragments containing podocyte-specific proteins, including podocalyxin and nephrin, into the urine, potentially before albuminuria onset [9,20]. Our finding that u-PDX correlates strongly with albuminuria but only modestly with eGFR suggests that u-PDX reflects ongoing podocyte injury rather than reduced filtering capacity. The inverse association with eGFR may arise from reduced nephron number and compensatory hyperfiltration, which increases mechanical stress on remaining podocytes. These mechanisms support the biological plausibility of u-PDX as an early DN marker.

### 4.3 Clinical implications

Our results suggest that u-PDX can complement existing clinical markers of DN. In our cohort, u-PDX appeared most informative in the earlier stages, particularly for distinguishing healthy controls from normoalbuminuric diabetic patients and normoalbuminuric patients from those with microalbuminuria, supporting the concept that u-PDX may capture subclinical podocyte injury before albuminuria-based staging fully reflects disease progression. Routine measurement could enable detection of high-risk individuals before microalbuminuria, facilitating earlier intervention—including tighter glycemic control, optimal blood-pressure management, dietary and lifestyle measures, and timely initiation of renin–angiotensin–aldosterone system inhibitors or sodium–glucose co-transporter-2 inhibitors, which are known to slow progression of diabetic kidney disease. Identifying such individuals earlier also creates an opportunity for nutritional and “food as medicine” strategies that may support metabolic and renal health [13]. u-PDX measurement is technically straightforward using ELISA, although standardization of assay protocols and reference values across laboratories is needed for clinical adoption.

### 4.4 Strengths and limitations

This study is among the first to evaluate u-PDX in a South Asian population and includes internal validation of diagnostic performance. Several limitations should be considered. First, the cross-sectional design precludes causal inference and assessment of prognostic value; u-PDX cannot be claimed to predict progression of diabetic nephropathy, and longitudinal follow-up is required to determine whether elevated u-PDX predicts future eGFR decline or progression to macroalbuminuria. Second, this was a single-center study with a relatively modest overall and subgroup sample size, which reduces the precision of sensitivity, specificity, and effect-size estimates; the wide confidence interval around the adjusted odds ratio reflects this and should be regarded as exploratory. Third, our findings may not generalize to other ethnicities, as previous studies report different baseline u-PDX levels and cut-offs (11, 12). Fourth, lipid profile, smoking, and alcohol use were not systematically captured, and metabolic comorbidities were not prespecified exclusions, leaving the possibility of residual confounding. Fifth, the ROC-derived thresholds were obtained from the same dataset in which discrimination was assessed; although bootstrap optimism correction showed little attenuation of AUC estimates (S1 Table), the reported AUCs, sensitivities, and specificities should be interpreted as exploratory and may overestimate performance in external populations, so external validation in an independent cohort is required before clinical application. Also, RAS inhibitors were an exclusion criterion to remove their confounding effects on albuminuria and podocyte dynamics, which was necessary to establish baseline diagnostic performance; because RAS inhibitors are standard of care for diabetic nephropathy, this exclusion limits the immediate real-world applicability of our findings, and future studies should include treated patients or adjust for treatment. Finally, We did not directly measure other podocyte or tubular markers, which limits the assessment of u-PDX’s relative performance; head-to-head comparison is needed in future work.

## 5. Conclusions

Urinary podocalyxin may serve as a valuable biomarker for early identification of diabetic nephropathy. u-PDX concentrations were higher in individuals with diabetes and increased progressively with albuminuria, correlating positively with uACR and inversely with eGFR. While the prognostic utility of u-PDX requires longitudinal investigation and the diagnostic estimates require external validation, these findings suggest that assessing podocyte injury via u-PDX could enhance early risk stratification and facilitate timely intervention in adults with T2DM.

### Generative AI statement

The authors affirm that Paperpal was used solely for language refinement and grammatical editing in this manuscript. All suggested revisions were reviewed and approved by the authors.

## Supporting information

S1 Table Multivariable Firth penalized logistic regression for the association between urinary podocalyxin and diabetic nephropathy among participants with type 2 diabetes (n  =  59; 30 events). Adjusted odds ratios are reported per 1 ng/mL increase in u-PDX and per unit for other covariates, with 95% confidence intervals. Firth’s penalized likelihood was used because quasi-complete separation produced unstable maximum-likelihood estimates.
(DOCX)

S2 Table Bootstrap optimism-corrected internal validation of receiver operating characteristic performance for urinary podocalyxin across stages of diabetic nephropathy. Apparent AUCs were calculated from the original dataset; internal validation used bootstrap optimism correction with 2,000 resamples. Optimism-corrected AUC = apparent AUC − mean optimism. Apparent cutoffs were determined by the Youden index, with sensitivity and specificity reported at the apparent cutoff; confidence intervals are 95% bootstrap intervals.
(DOCX)
