# Supplementary material for: Urinary podocalyxin as an early biomarker for diabetic nephropathy
Source: PLoS One. 2026 Jul 23;21(7):e0347975. doi: 10.1371/journal.pone.0347975 (PMC13395460; doi:10.1371/journal.pone.0347975)
Supplement: S1 Table — Adjusted odds ratios are reported per 1 ng/mL increase in u-PDX and per unit for other covariates, with 95% confidence intervals. Firth’s penalized likelihood was used because quasi-complete separation produced unstable maximum-likelihood estimates. (DOCX) [file pone.0347975.s001.docx]

**S1 Table.** Multivariable Firth penalized logistic regression for the association between urinary podocalyxin and diabetic nephropathy among participants with type 2 diabetes mellitus.

| **Variable** | **Adjusted OR** | **95% CI** | **p-value** |
| --- | --- | --- | --- |
| u-PDX (per 1 ng/mL) | 22.67 | 2.47 – 208.09 | 0.006 |
| Age (per year) | 0.92 | 0.78 – 1.08 | 0.30 |
| Male sex | 0.09 | 0.01 – 1.52 | 0.095 |
| Duration of diabetes (per year) | 1.04 | 0.84 – 1.29 | 0.74 |
| Systolic blood pressure (per mmHg) | 0.89 | 0.79 – 1.01 | 0.066 |

Outcome: diabetic nephropathy (microalbuminuria or macroalbuminuria) versus normoalbuminuria among participants with type 2 diabetes (n = 59; 30 events). The model was adjusted for age, sex, duration of diabetes, and systolic blood pressure. Because the strong separation between groups produced unstable maximum-likelihood estimates (quasi-complete separation), the model was fitted using Firth’s penalized likelihood. Adjusted odds ratios for u-PDX are expressed per 1 ng/mL increase. The wide confidence interval for u-PDX reflects the modest sample and event count; the estimate should be interpreted as exploratory. OR, odds ratio; CI, confidence interval; u-PDX, urinary podocalyxin.
