# Supplementary material for: Urinary podocalyxin as an early biomarker for diabetic nephropathy
Source: PLoS One. 2026 Jul 23;21(7):e0347975. doi: 10.1371/journal.pone.0347975 (PMC13395460; doi:10.1371/journal.pone.0347975)
Supplement: S2 Table — Apparent AUCs were calculated from the original dataset; internal validation used bootstrap optimism correction with 2,000 resamples. Optimism-corrected AUC = apparent AUC − mean optimism. Apparent cutoffs were determined by the Youden index, with sensitivity and specificity reported at the apparent cutoff; confidence intervals are 95% bootstrap intervals. (DOCX) [file pone.0347975.s002.docx]

Supplementary Table S2: Bootstrap optimism-corrected internal validation of receiver operating characteristic performance for urinary podocalyxin across stages of diabetic nephropathy.

Apparent AUC values were calculated from the original dataset. Internal validation was performed using bootstrap optimism correction with 2000 resamples. Mean optimism was defined as the average difference between bootstrap sample performance and performance tested against the original sample. Optimism-corrected AUC was calculated as the apparent AUC minus the mean optimism. The apparent cutoff for each comparison was determined using the Youden index. Sensitivity and specificity are reported at the apparent cutoff. Confidence intervals are presented as 95% bootstrap intervals. ROC comparisons were performed for healthy controls versus diabetic patients without albuminuria, diabetic patients without albuminuria versus those with microalbuminuria, and microalbuminuric versus macroalbuminuric diabetic patients.

| **comparison** | **n_total** | **n_negative** | **n_positive** | **apparent_auc** | **apparent_auc_ci_low** | **apparent_auc_ci_high** | **bootstrap_mean_optimism** | **optimism_corrected_auc** | **corrected_auc_ci_low** | **corrected_auc_ci_high** | **apparent_cutoff** | **sensitivity** | **specificity** | **boot_resamples_used** | **sample_sizes** | **apparent_95_CI** | **corrected_95_CI** |
| --- | --- | --- | --- | --- | --- | --- | --- | --- | --- | --- | --- | --- | --- | --- | --- | --- | --- |
| **Healthy vs Diabetes without albuminuria** | 57 | 28 | 29 | 0.989 | 0.9599929667519180 | 1.0 | 0.0 | 0.989 | 0.9778325123152710 | 1.0 | 0.8 | 100.0 | 96.4 | 2000 | 28 vs 29 | 0.96 to 1.0 | 0.978 to 1.0 |
| **Diabetes without albuminuria vs Microalbuminuria** | 44 | 29 | 15 | 0.918 | 0.7923921130952380 | 1.0 | 0.003 | 0.915 | 0.8367816091954020 | 1.0 | 1.6 | 86.7 | 93.1 | 2000 | 29 vs 15 | 0.792 to 1.0 | 0.837 to 1.0 |
| **Microalbuminuria vs Macroalbuminuria** | 30 | 15 | 15 | 0.893 | 0.7366071428571430 | 1.0 | 0.0 | 0.893 | 0.7866666666666670 | 1.0 | 5.51 | 86.7 | 93.3 | 2000 | 15 vs 15 | 0.737 to 1.0 | 0.787 to 1.0 |
